# A comparison of methods used to unveil the genetic and metabolic pool in the built environment

**DOI:** 10.1186/s40168-018-0453-0

**Published:** 2018-04-16

**Authors:** Cinta Gomez-Silvan, Marcus H. Y. Leung, Katherine A. Grue, Randeep Kaur, Xinzhao Tong, Patrick K. H. Lee, Gary L. Andersen

**Affiliations:** 1 Department of Environmental Science, Policy, and Management, University of California, Berkeley, CA USA; 2 Environmental Genomics and Systems Biology Division, Lawrence Berkeley National Laboratory, Berkeley, CA USA; 3 School of Energy and Environment, City University of Hong Kong, Tat Chee Avenue, Kowloon, Hong Kong; 4 Current affiliation: Department of Physical Therapy and Rehabilitation Science, University of California, San Francisco, CA USA

**Keywords:** DNA, RNA, Indoor microbiome, Surface, Air, Sample storage, RNAStable, Extraction kit

## Abstract

**Background:**

A majority of indoor residential microbes originate from humans, pets, and outdoor air and are not adapted to the built environment (BE). Consequently, a large portion of the microbes identified by DNA-based methods are either dead or metabolically inactive. Although many exceptions have been noted, the ribosomal RNA fraction of the sample is more likely to represent either viable or metabolically active cells. We examined methodological variations in sample processing using a defined, mock BE microbial community to better understand the scope of technique-based vs. biological-based differences in both ribosomal transcript (rRNA) and gene (DNA) sequence community analysis. Based on in vitro tests, a protocol was adopted for the analysis of the genetic and metabolic pool (DNA vs. rRNA) of air and surface microbiomes within a residential setting.

**Results:**

We observed differences in DNA/RNA co-extraction efficiency for individual microbes, but overall, a greater recovery of rRNA using FastPrep (> 50%). Samples stored with various preservation methods at − 80°C experienced a rapid decline in nucleic acid recovery starting within the first week, although post-extraction rRNA had no significant degradation when treated with RNAStable. We recommend that co-extraction samples be processed as quickly as possible after collection. The in vivo analysis revealed significant differences in the two components (genetic and metabolic pool) in terms of taxonomy, community structure, and microbial association networks. Rare taxa present in the genetic pool showed higher metabolic potential (RNA:DNA ratio), whereas commonly detected taxa of outdoor origins based on DNA sequencing, especially taxa of the *Sphingomonadales* order, were present in lower relative abundances in the viable community.

**Conclusions:**

Although methodological variations in sample preparations are high, large differences between the DNA and RNA fractions of the total microbial community demonstrate that direct examination of rRNA isolated from a residential BE microbiome has the potential to identify the more likely viable or active portion of the microbial community. In an environment that has primarily dead and metabolically inactive cells, we suggest that the rRNA fraction of BE samples is capable of providing a more ecologically relevant insight into the factors that drive indoor microbial community dynamics.

**Electronic supplementary material:**

The online version of this article (10.1186/s40168-018-0453-0) contains supplementary material, which is available to authorized users.

## Backgound

The majority of individuals from the developed world spend over 90% of their time indoors, or in other built environments (BEs) [1], which now collectively represent approximately 0.5% of the world’s total terrestrial area [2]. At the same time, indoor occupants co-exist with a diverse community of microorganisms, termed the BE microbiome, predominantly constituted of bacteria and fungi. While most members of this community are commensal in nature, some may be associated with adverse health outcomes [3, 4]. Thus, understanding the structure of the BE microbiome, how it is affected by different factors, and how the microbiome affects occupant health, is of utmost importance in safeguarding the comfort and well-being of modern individuals.

While earlier culture-based investigations have provided information on the viable and cultivable components of the indoor microbiome, high-throughput sequencing (HTS) of the DNA of the 16S ribosomal RNA gene (rRNA gene) offered unparalleled insights into the breadth of the diversity and composition of the BE microbiome. Studies have pointed to the outdoor environment, occupancy, and building characteristics (i.e., ventilation) as the main sources of the indoor microbial community [5–7]. Although rRNA gene sequences from isolated genomic DNA (gDNA) provide a glimpse of the genetic potential of a microbial ecosystem, targeting gDNA impedes the differentiation of viable from non-viable components of the microbial assemblage [8, 9]. gDNA detected via HTS may originate from dead and inactive cells, or from extracellular DNA captured in air or deposited onto surfaces. Demonstrations of viability in bioaerosols have been limited to cultivation-based techniques conducted in laboratories, which are neither comprehensive nor representative of the atmosphere in which these microbes are captured [10]. More recently, culture-independent methods based on membrane integrity have demonstrated that the majority of gDNA detected in a BE may actually come from dead cells or those with a compromised cell membrane [11, 12], and that approximately only a 10% of the bacteria in the human skin, an important source of the BE microbiome, are active [13]. Moreover, gDNA-based and viable community comparisons reveal differences in the taxonomies, microbial diversities, and/or compositions of the respective assemblages within indoor cleanroom environments [11, 12, 14]. Although a greater taxonomic diversity is observed when compared to culture-based approaches, this method has limitations related to the variability in microbial membrane or wall structures and sample treatment optimization [15].

Alternatively, the direct examination of rRNA through RNA isolation has been found, in general, to be a more reliable indicator of cellular viability than rRNA gene targets [16–19]. Under stress or starvation, cellular endonuclease(s) may initiate functional ribosome degradation, whereas RNase I homologs have been demonstrated to degrade ribosomes in physically damaged or dying bacterial cells [20]. This relatively labile property of cellular ribosomes has been used in numerous environmental studies to better assess the active and viable component of the community, as well as elucidating the functional relevance of rare taxa [15, 21–25].

With limited biomass, the BE poses a special challenge in providing sufficient material for RNA isolation, storage and analysis. Selective pressures imposed by the BE, such as desiccation and UV irradiation, generate additional challenges in extracting intact nucleic acids. Creating a robust and reproducible method for nucleic acid extraction, especially the RNA fraction, is essential to more accurately infer the survival and adaptive potentials of indoor microbes, and the viability of pathogens that are potentially present in BEs. Moreover, this will empower BE scientists to determine how the building and occupant attributes potentially shape the viable components of the BE microbiome.

**Fig. 1:**
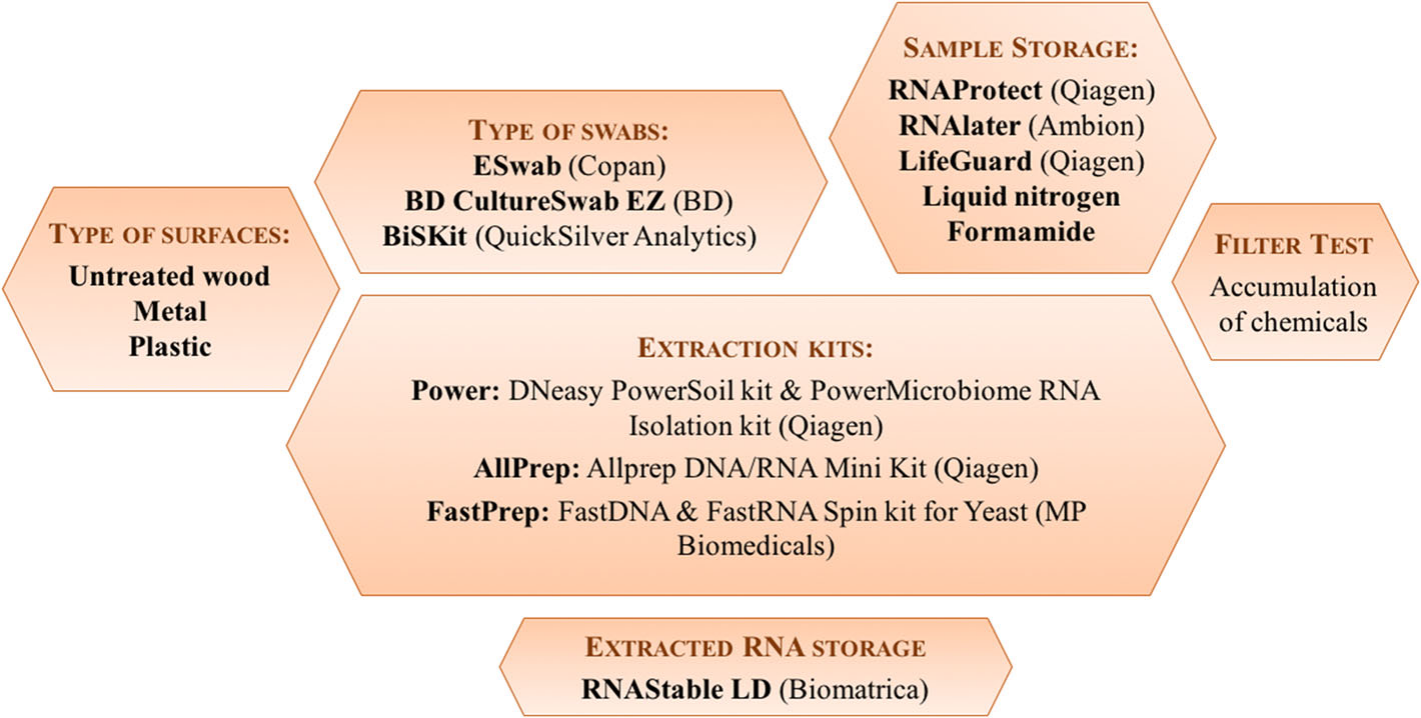
Schematic chart of in vitro workflow organized by sequence of tasks involved in sampling and extraction. Multiple stages of the in vitro sampling and extraction processes (types of swabs, and surfaces, sample storage prior to extraction, extraction method, and nucleic acid preservation) were tested for the optimal methods in terms of nucleic acid recovery

Variations in methodologies, such as sample collection, storage, or commercial extraction kits, have been shown to have an impact on microbiome data interpretation [26–30]. Following sample extraction, nucleic acids, in particular RNA molecules, are susceptible to stochastic and rapid degradation, thereby introducing bias in the detected community [15]. Efforts towards method standardization will increase the validity of future inter-laboratory comparative investigation. In the first part of the study, we tested and analyzed the efficiency of different sample collection methods, type of surfaces, sample and nucleic acids preservation methods, and DNA/RNA co-extraction kits, quantifying the proportion of the spiked low biomass mock community recovered in each step. In the second part, we tested the applicability of the genetic (DNA) and metabolic pool (RNA) study to air and surface samples of a residential unit, analyzing community through rRNA gene and rRNA HTS. We show the variable performance characteristics of four common types of indoor microbes using three different DNA/RNA co-extraction kits, three different sampling swabs and surface types, six different sample storage methods, and tested the performance of a room temperature RNA storage method (Fig. 1). We used one of these methods for a detailed analysis of the indoor air and surfaces of a single family residential unit. Differences in microbial community composition between the DNA and rRNA fraction of the BE samples suggests that the potentially viable or metabolically active portion of the microbial community (rRNA fraction) may provide a more accurate view of the ecological factors that drive the indoor microbial community dynamics when compared with the more stable DNA fraction.

## Methods

A mock community including pure bacterial and fungal strains were included to test the effects of different DNA/RNA co-extraction methods, swab and surfaces types, and sample and nucleic acid storage conditions, over the recovery rate of both components (DNA and RNA). In vitro recovery rate of nucleic acid material was based on the result of quantitative PCR (qPCR). Based on in vitro results, sequencing analysis was performed on air and surface samples collected from an unoccupied bedroom to compare the total genetic pool and the viable components of the microbial communities. The following sections describe in detail the different tests conducted.

### Pure strains and mock community

Four pure strains representing different types of cells were used: *Pseudomonas syringae*, *Bacillus subtilis*, *Saccharomyces cerevisiae*, and *Aspergillus nidulans.* (Additional file 1: Table S1). The pure strains were grown in liquid media to mid-log phase, and then the concentration of cells was measured by optical density prior to dilution in PBS buffer. The mock community was prepared using 35% of each type of bacteria (*P. syringae* and *B. subtilis*), 20% of yeast (*S. cerevisiae*), and 10% of fungi (*A. nidulans*). Microbial isolate proportions were based on recent indoor dust studies that quantified the bacterial and fungal community in BE samples [31–33]. For each experimental set, a final cell density of ca. 10^4^ cells/mL was spiked onto the samples, and the samples were extracted along with an extraction blank and an aliquot of the dilutions in PBS buffer as a reference of the actual number and RNA content of the spiked cells.

### qPCR

Specific primers targeting the 16S and 18S rRNA genes were designed for each strain using primer-BLAST [34] (Additional file 1: Table S1). The specificity of each primer set was verified to confirm no cross-amplification. Replicated samples and dilutions, as well as a no template, negative control were quantified on a iCycler Thermal Cycler and MyiQ™ Single-Color Real-Time PCR Detection System (BioRad, Hercules, CA, USA) (Additional file 2: Table S2). The qPCR standards and their cycle threshold (Ct) values were also used as a positive control, with standard deviation < 1.5 from the average Ct value. The Ct values of the no template, negative controls were, at minimum, 5 cycles higher than the detection limit (Ct value of the most diluted qPCR standard) [35].

The qPCR standards were constructed amplifying the rRNA genes from the pure strains using the designed primers and the same quantification protocol (Additional file 1: Table S1 and Additional file 2: Table S2) with no EvaGreen and no melting curve. After purification (QIAquick PCRPurification Kit, Qiagen, Hilden, Germany), the amplicons were cloned and transformed into *Escherichia coli* TOP10 using the Zero Blunt TOPO PCR Cloning Kit (Invitrogen, Waltham, MA, USA). The cloned sequences were confirmed by Sanger sequencing at UC Berkeley DNA Sequencing Facility. Plasmids were extracted and purified (QIAprep Spin Miniprep Kit, Qiagen, Hilden, Germany), then linearized (BamHI restriction enzyme, New England BioLabs Inc., Ipswich, MA, USA) followed by the quantification of the DNA concentration (Qubit® fluorometer and Qubit® dsDNA HS Assay Kit, Invitrogen, Waltham, MA, USA) and preservation at − 20 °C.

### RNA sample processing

Due to the labile nature of the RNA, and to avoid introducing undesired bias, RNA was extracted, digested with DNase followed by reverse-transcription (RT), and quantified on the same day. Following extraction by one of the three evaluated protocols, the RNA was digested with the TURBO DNA-free Kit (Ambion, Thermo Fisher Scientific, Waltham, MA, USA). Reverse transcription of the isolated RNA into complementary DNA (cDNA) followed manufacturer’s recommendations (Additional file 2: Table S2). To assess for DNA contamination during the extraction process, RNA was also subjected to parallel reactions without reverse transcriptase (RT negative control).

### Co-extraction protocol tests

Approximately 10^4^ cells of the previously described BE mock community were directly spiked on eSwabs (a nylon fiber tipped swabs that is commonly used in indoor studies; Additional file 3: Table S3) and extracted along with the reference sample, in duplicate. Three different kit sets were selected (Additional file 3: Table S3) and optimized for DNA and RNA co-extraction as detailed in Additional file 4: Text S1. The Power co-extraction protocol first involved the DNeasy PowerSoil Kit, extensively used for indoor microbial samples (i.e., [27, 36, 37]) and continued with the PowerMicrobiome RNA Isolation Kit as part of a co-extraction, the AllPrep DNA/RNA Mini Kit is designed for co-extraction and has been successfully used for low biomass samples (i.e., [38]), and the FastDNA and FastRNA SPIN kit for Yeast were selected as a third co-extraction method to test.

### Filter test

To obtain sufficient biomass from indoor air for DNA or RNA analysis, several hours of sample collection time onto filters from indoor mirobiological air samplers is typically required. During this time, interfering chemicals and abiotic particles accumulate along with the desirable biomass [39]. We evaluated the extraction rate and the potential interference of the chemical and particles accumulated on the air filters over the DNA/RNA co-extraction. Sterilized and clean cellulose nitrate filters (diameter, 25 mm; pore size, 0.2 μm; Whatman, Maidstone, UK) that had not been subjected to air sampling, or filters used to collect air samples as described below for indoor residential samples [37, 40], were spiked with approximately 10^4^ cells/mL of pure culture. Duplicated spiked filters were extracted, along with the reference sample, using FastPrep co-extraction protocol, and were processed and quantified as described above.

### Swab and surface test

Swab and surface types were examined to evaluate their sampling performance. For surfaces, three physically diverse surface types commonly found in the BE (plastic, metal, and untreated wood) were evaluated. Also, three types of swabs (eSwab, BBL CultureSwab EZ, and BiSKit) were compared (Additional file 3: Table S3). The eSwab is a nylon fiber tipped swabs with inorganic buffer commonly used in indoor studies [36, 41], BBL CultureSwab EZ is a polyurethane-tipped fiber swabs that was found to have superior performance in human microbiome sampling [42], and BiSKit is an sponge-based method with inorganic buffer, commonly used for sampling larger surfaces [43].

The mock community diluted in PBS buffer was spiked on a 30 cm^2^ of each type of surface previously washed and sterilized. Preliminary tests evaluating the surface sterilization were conducted, with no amplification detected for any of the primer sets. After the surface was completely dried, it was dry-swabbed in two perpendicular directions. 1 mL of PBS buffer was added to the BBL CultureSwab EZ, and the default buffers were used for eSwab and BiSKit sampling kits. The eSwab and the BBL CultureSwab EZ were then vortexed for 2 min, transferring just the buffer to the Lysis Matrix Y from the FastPrep co-extraction protocol (Additional file 4: Text S1). The manufacturer’s instructions were followed for BiSKit, centrifuging the buffer for 15 min. at 6800×*g* to pellet the sample, discarding the buffer, and leaving only 1 mL to resuspend the sample and proceed with the FastPrep co-extraction protocol along with the reference sample. All samples were duplicated.

### Sample storage test

Six sample storage conditions were tested (Additional file 3: Table S3) including three different commercial solutions designed to preserve the RNA molecules, one tested at two different storage temperatures; formamide, proven to inhibit the action of enzymes stabilizing the extracted RNA from degradation [44]; and flash freezing with liquid nitrogen, the most commonly used method.

1 mL of the mock community were aliquoted into 2 mL microcentrifuge tubes, were centrifuged 5 min at 23,000×*g* to pellet the cells, and were preserved under different conditions, following the manufacturer’s instructions. Briefly, the three commercial solutions (RNAlater, RNAProtect, and LifeGuard Preservation Solution) were used as recommended by the manufacturers (Additional file 5: Text S2). Some pellets were covered with formamide, and some were flash frozen in liquid nitrogen and stored. Triplicate samples were extracted at five different time points spanning 3 months of storage. The FastPrep co-extraction protocol was used, and the samples were processed and quantified as described above.

### Extracted nucleic acids preservation test

In order to evaluate independently the performance of the RNAStable LD post-extraction (Additional file 3: Table S3) with low biomass samples, RNA from a mock community was extracted following the FastPrep co-extraction protocol. The extracted RNA was then aliquoted and preserved with RNAStable LD, drying the samples in a concentrator and preserving them in the sealed moisture barrier foil bag according with the manufacturer’s instructions. At different time points spanning 3 months of storage, triplicated samples were rehydrated for 15 min and then processed and quantified as described above.

### Indoor residential sampling

Indoor residential samples were collected during April 2017 in an approximately 10 m^2^ bedroom of a single-occupancy residential unit in Hong Kong [37, 40]. Bioaerosols were collected onto cellulose nitrate filters as described previously [37, 40] using the Leland Legacy portable pumps (SKC Inc., Eighty Four, PA, USA), each at a flow rate of 9 l/min. Filtering a total of 4.32 m^3^ air per sample and a Sioutas Cascade Impactor (SKC Inc., Eighty Four, PA, USA) with a D-plate accelerator (collects particles with a diameter larger than 0.25 μm). All windows were closed, and the room was left unoccupied during sampling (except when required to change sampling filters and disinfect the apparatus). To minimize the effect of sampling time on differences in microbial community composition [36], all the airborne samples were collected within 24 h with four pumps running in parallel at three 8-h shifts (00:00–08:00, 08:00–16:00, and 16:00–00:00), pooling together one filter from each of the three sampling shifts. Different surfaces located at different distances from the bioaerosol pumps were swabbed for 15 s using eSwab after the air samples were collected. The surface samples included wooden bed side (~ 30 cm from air samplers) and front rims (~ 180 cm from air samplers), desk chair plastic surface (~ 90 cm from air samplers), nearby window stone surface (indoor, ~ 200 cm from air samplers), and an outdoor cement surface (outdoor, ~ 210 cm from air samplers). Air filters and swabs for RNA extraction (but not DNA) were immediately submerged in 30 μL of LifeGuard Preservation Solution, substituting the inorganic buffer form the eSwabs. All the samples were stored at − 80^°^*C* until nucleic acid extraction.

### Indoor samples processing

gDNA from the indoor surface and air samples were extracted using the DNeasy PowerSoil Kit with slight modifications as previously described [37]. Based on the in vitro results obtained for rRNA extraction, RNA from air samples was extracted using the FastRNA SPIN Kit for Yeast with slight modifications as described in Additional file 4: Text S1. Prior to extraction, all surface samples were vortexed for 2 min and the swab discarded. All samples were then centrifuged at 23,000×*g* 15 min and the LifeGuard discarded. The pellet was resuspended with the appropriate lysis buffer and proceed with the extraction. To assess the effect of sample preservation and storage time on environmental BE samples, DNA and RNA from air filter samples were extracted on six different time points spanning 6 weeks of storage.

RNA from environmental samples was processed as described above. gDNA and cDNA were subjected to bacterial PCR by primer pairs targeting the 16S rRNA V4 (Additional file 1: Table S1), with thermal cycling conditions as described previously [37]. Triplicate-pooled PCR reactions from each sample were sent to Seqmatic (Fremont, CA, USA) for sequencing library preparation and sequence analysis on the MiSeq platform.

### Indoor samples bioinformatics analysis

A total of 1,337,415 bacterial 16S rRNA paired end sequences were analyzed using the Quantitative Insights Into Microbial Ecology (QIIME v. 1.9) pipeline [45]. The raw forward and reverse paired sequence reads were assembled and quality filtered with USEARCH (version 10.0.240) [46], discarding the reads with a total expected error of greater than 1 and shorter than 280 bases. Following quality filtering, a total of 914,008 sequences were clustered into operational taxonomic units (OTUs) using the UPARSE [47], with a clustering identity threshold of 97%. Taxonomy classifications were performed with the SILVA [48] as reference database (version 128 release, 97% representative set file, total of 166,393 sequences). Chimeric OTUs were identified using UCHIME2 [49] using the SILVA database. Negative controls of different sample groups (controls for each of DNA and RNA extraction) were included, and OTUs of taxonomic lineages present in more than 3% in the controls were removed from all samples. Following chimeric, contaminating, chloroplast, and mitochondrial OTU removal, OTUs present in less than 100 reads of the entire dataset were removed from the dataset to reduce the effect of noise on data analysis. Thus, a total of 569,372 reads were included for microbial community analyses. Community membership and composition were analyzed using unweighted and weighted UniFrac distances, respectively [50]. SParse InversE Covariance Estimation for Ecological Association Inference (SPIEC-EASI) was used to assess potential ecological associations between microbial taxa in the active and total populations, with a minimum lambda ratio of 0.01, and reiteration of 50 times [51]. Network structural properties, including degree distribution and natural connectivity in response to node removal, were examined using R [52]. Network visualization was constructed with Cytoscape (version 3.5.0) [53]. To look at the microbiome overlap between the viable bioaerosol population and viable populations of nearby surfaces, Bayesian source-tracking [54] approach was performed in QIIME to estimate the contribution of potential sources of the viable component of the residential microbiome. The RNA-based community from different surface at various distances from the air sampling pumps were included in analysis. We performed source-tracking analysis based on two possible scenarios: (1) microbes be re-suspended into the air from surfaces (i.e., air as microbiome sink, and surfaces as sources), and (2) microbes be settled onto nearby surfaces from the air (i.e., air as source, and surfaces as sinks).

### Statistical analyses

The results of the in vitro test are expressed as the proportion of the DNA (as 16S/18S rRNA gene copies) and the RNA (as 16S/18S rRNA copies) recovered from the spiked samples in comparison with the reference sample of each set of experiments. R software [55] was used for the analyses, with ggplot2 package [56] for generating the plots. Nonparametric Kruskal-Wallis (KW) and Mann-Whitney (MW) tests were employed and *p* values were adjusted for multiple comparisons using the false discovery rate (FDR).

ANOSIM Global R and PERMANOVA pseudo-F statistics were calculated for the indoor microbiome samples using QIIME, based on the default setting of 999 permutations. To identify differentially abundant OTUs between genetic and metabolic pool, DeSeq2 was performed with an adjusted *p* < 0.05 considered statistically significant. Only OTUs with DeSeq2 log-fold changes of at least |2| were considered to be differentially abundant. Where indicated, *p* values were adjusted for multiple comparisons using the FDR, and Kendall’s τ ranked correlation was computed in R [52].

## Results and discussion

### Evaluation of sample preparation and storage

Molecular-based methods have greatly increased our understanding of the diversity of ecological interactions observed among the members of the BE microbial community when compared to culture-based methods. However, discrepancies in the conclusions based on microbial community composition and inferred metabolic activity among different studies have highlighted the need to better understand how the various sample preparation methods influence an individual study’s results. In this study, we compared the efficiencies of several common DNA/RNA sample co-extraction methods, materials for surface sampling on different surfaces, and sample and nucleic acid storage methods.

**Fig. 2:**
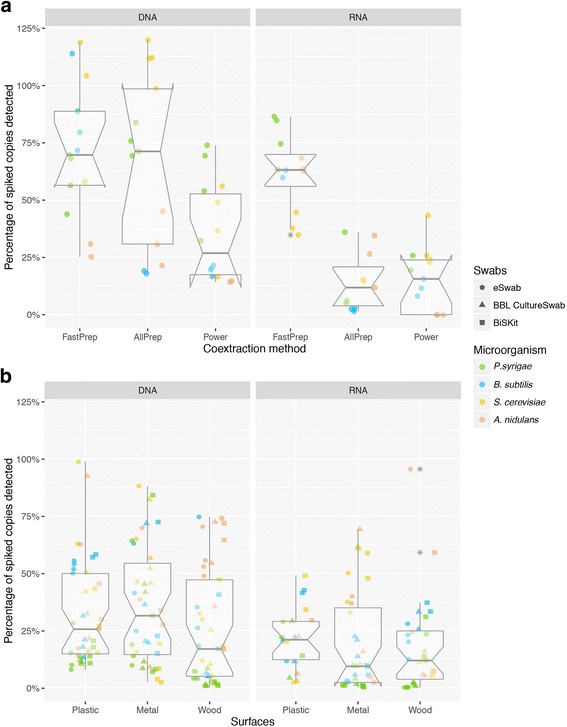
In vitro DNA/RNA co-extraction efficiencies tests. **a** Co-extraction protocol comparison. FastPrep: co-extraction protocol using the FastDNA and FastRNA SPIN Kit for Yeast; AllPrep: using the AllPrep DNA/RNA Mini Kit; Power: using DNeasy PowerSoil Kit and PowerMicrobiome RNA Isolation Kit. FastPrep method was the most efficient co-extraction method. **b** Comparison of the recovery efficiency from different type of surfaces (plastic, metal, and untreated wood trays) using different swabs (eSwab, BBL Culture Swab, and BiSKit) and the FastPrep co-extraction protocol. No significant differences were detected between swabs or sampled surfaces. The fungi had typically higher recovery rates than bacteria while *P. syringae* consistently had the lowest recovery for both nucleic acids

Log-phase cultures of *P. syringae*, *B. subtilis*, *S. cerevisiae*, and *A. nidulans* with final cell densities of ca. 10^4^ cells/mL were used to evaluate the DNA/RNA co-extraction efficiencies of the DNeasy PowerSoil Kit and PowerMicrobiome RNA Isolation Kit (Power), the AllPrep DNA/RNA Mini Kit (AllPrep) and the FastDNA and FastRNA SPIN Kit for Yeast (FastPrep), all with minor modifications as stated in Additional file 4: Text S1. Considerable variation in the efficiency of co-extraction between microorganisms was observed when comparing the three methods as well as, in most cases, within each method (Fig. 2a). The FastPrep method was significantly more efficient in RNA extraction for all microorganisms compared to the other two methods (*p* < 7×10^− 5^) although no statistically significant differences were found among the three methods in DNA extraction. The Power Kit appeared to have slightly less variation in efficiency between microorganism type for DNA and one of the lower overall extraction efficiencies for RNA compared to the other kits.

Within each method, differences in extraction efficiency were observed for individual microbes when comparing DNA to RNA. For example, the yeast *S. cerevisiae* was among the highest in recovery of DNA and the lowest for RNA (*p* < 0.1) when using the FastPrep Kit. Conversely, the filamentous fungi *A. nidulans* had one of the lowest efficiencies of recovery for DNA from the AllPrep Kit, but one of the highest efficiencies for RNA. We believe that this is due to the very large method variation that was observed, rather than in intrinsic differences in RNA copy number characteristic of each species. This observed variability in nucleic acid extraction efficiency indicates that it is important to understand the range of technical variation in setting a threshold for what is a significant difference in inferring metabolic activity/viability using RNA:DNA ratios.

Cellulose nitrate filters from the air sampler were spiked with the BE mock community, followed by nucleic acid co-extraction, to evaluate the potential interference with chemicals accumulated on the filters during the air sampling. No significant differences between the clean and used filters spiked with microorganisms were detected (MW *p* > 0.07), showing a similar DNA and RNA extraction efficiency to the obtained for the spiked swabs extracted with the same protocol, FastPrep (MW *p* = 0.67).

We also determined cell recovery efficiency from three different types of spiked surfaces when compared with the direct application of the mock community to three different types of swabbing material (Fig. 2b). Using the FastPrep protocol for DNA/RNA co-extraction, we found that there was very little difference in the recovery of microorganisms among the three different surface swabs that were tested. There was considerable variability in the recovery of the mock BE community when swabbed from the three different surfaces, resulting in no statistically significant difference in performance in either DNA or RNA recovery based on surface type. Within the BE mock community, the fungi had typically higher recovery rates than bacteria, while *P. syringae* consistently had the lowest recovery for both DNA and RNA. One potential explanation for this could be the robustness of the cell wall, with fungi generally possessing more environmentally resistant cell walls than gram-negative bacteria.

**Fig. 3:**
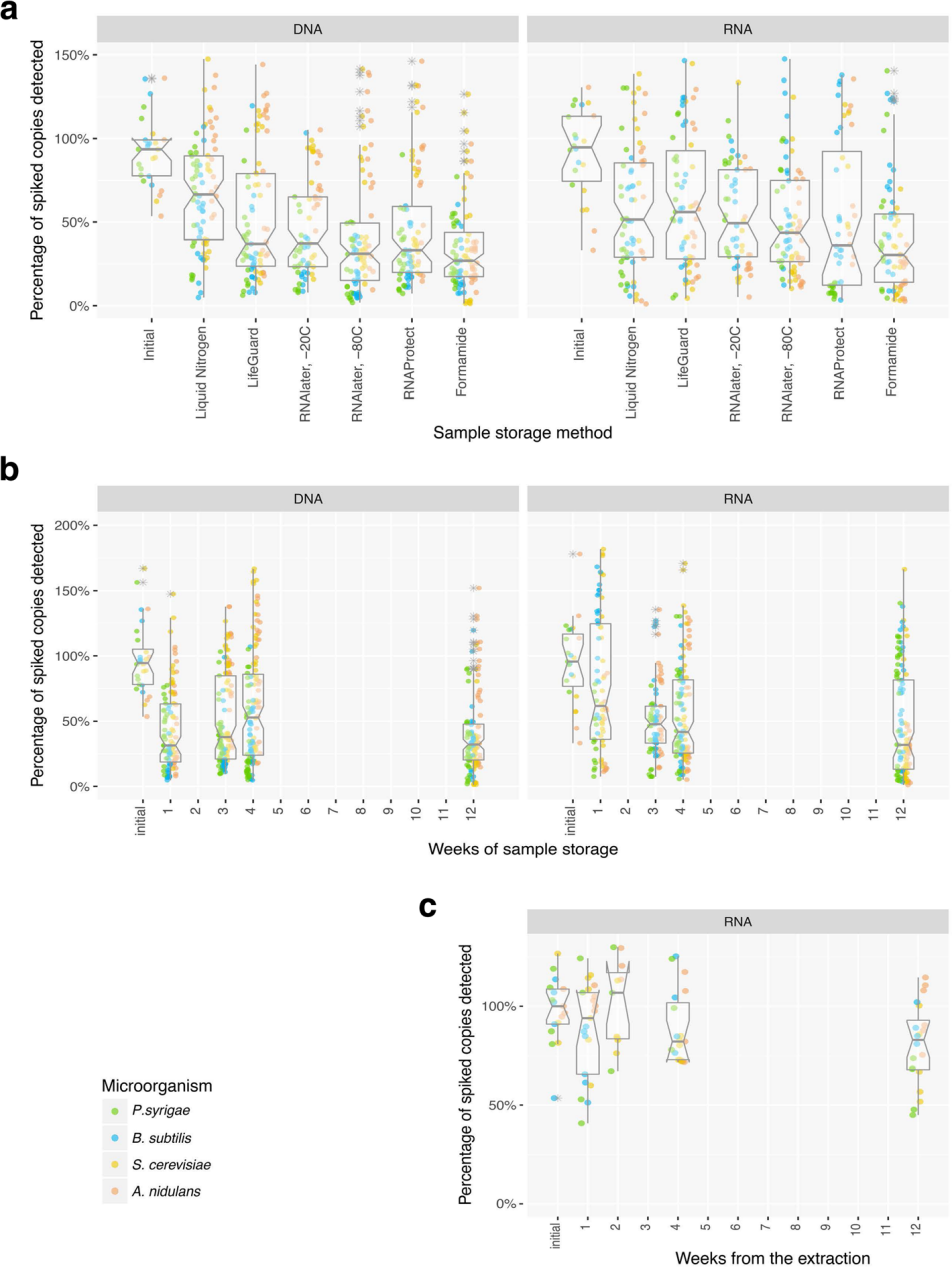
In vitro storage tests. Evaluation of the nucleic acid recovery efficiency and stability after 3 months storage period using the FastPrep co-extraction protocol. **a** Comparison of the recovery efficiency of the six methods preserving the samples prior nucleic acid extraction. **b** Evolution of the nucleic acid recovery efficiency over time of the preserved samples prior nucleic acid extraction. Both DNA and RNA stability rapidly decline in all the storing methods, with the liquid nitrogen the best option preserving samples for DNA extraction. **c** Stability over time of the extracted RNA stored in RNAStable at room temperature. The solution preserved virtually intact the extracted RNA over the 3-month storage period

BE microbiome studies typically require an extensive sampling regime over multiple locations and/or time points. To avoid extensive degradation of the nucleic acids prior to analysis, samples are stored in a way to optimize their integrity. We evaluated six different methods for sample storage preserving the DNA and RNA and an additional method specifically for post-extracted RNA. The two most striking findings were the very high level of variability in replicate performance of the nucleic acid preservation within each method (Fig. 3a), and the rapid decline in recovery starting at the first week (Fig. 3b). Within this background of high variability and rapid decline in recovery, flash freezing with liquid N_2_ had a trend towards slightly higher recovery of DNA with an average of ca. 71% of the DNA (MW *p* < 1 ×10^− 3^)_._ None of the preservation methods outperformed the others in the storage of RNA. This high level of intraspecies variability should be of concern when setting up an experimental design for a BE study so that, whenever possible, immediate nucleic acid extraction should be attempted.

An additional commercially available solution that preserves extracted RNA, RNAStable, was also evaluated. Tests with the BE mock community demonstrated post-extraction RNA preservation during the 3-month storage with no significant degradation (Fig. 3c). The RNAStable solution was relatively easy to use. Samples were dried in tubes and stored at ambient temperature. The same company provide a similar product designed for the extracted DNA storage in the same conditions, DNAStable, proven to work for more than year-long storage [57, 58]. Dry-storing the nucleic acids has a great potential by reducing shipment, space, and energy costs while reducing the carbon footprint.

Although microbes that are found in indoor air and surfaces commonly originate from external sources, their ability to persist is dependent on their ability to survive environmental stresses, such as low humidity, UV light, and lack of nutrients. Identification of the indoor microbial community composition and abundance through DNA-based methods does not provide the ability to distinguish the majority of the microbial community that is either metabolically inactive or non-viable from the minority that remains viable. Even with the previously mentioned caveats of using the more labile RNA to identify potentially viable or active microbes, it may still provide more ecologically relevant information than DNA when a majority of the microbes are dead or dormant. With multiple replicates, we identified very high levels of variation in all steps of process of BE samples for analysis. In many cases, we found that the variation in response among different organisms was greater than the different commonly used methods, leading us to conclude that standardization of methods among BE researchers may not produce the desired clarity that is hoped by its proponents. A practical solution may be to use this information to attempt to minimize variation in sample preparation and storage wherever possible, and to require that the differences among samples be greater than the observed differences found within the methods for a biologically meaningful conclusion. As a test case, we examined the rRNA and DNA nucleic acid fractions of surface and air samples in a residential unit. By examining differences in the potentially active/viable subset in comparison with the total BE microbial community, we were interested in determining if the rRNA fraction would add value to the more commonly used, DNA-based, microbial analysis.

### Characterization of genetic and metabolic pool components in residence unit

**Fig. 4:**
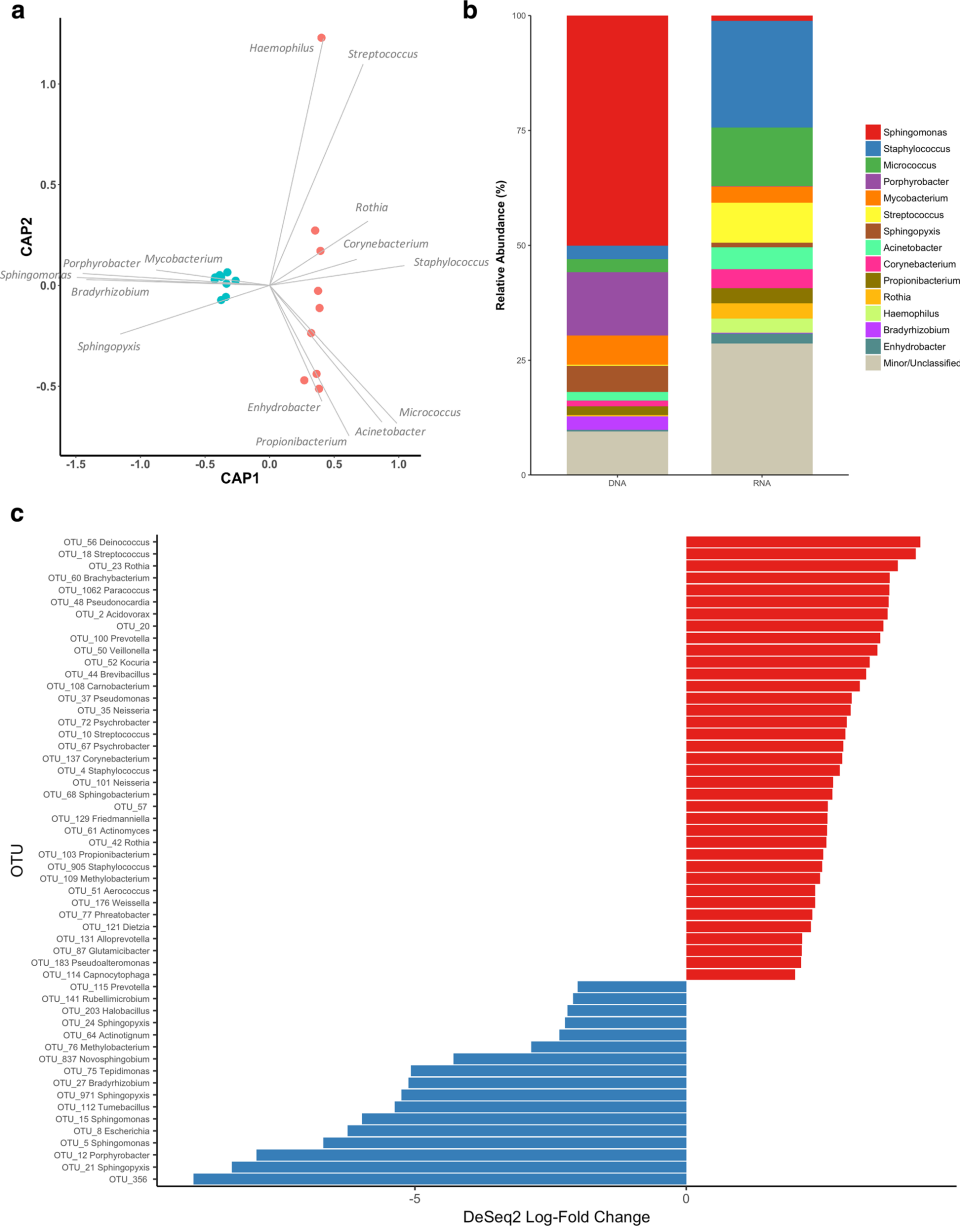
Differences in the DNA and RNA components of the indoor air microbiome. **a** Distance-based redundancy analysis of community composition as measured by weighted UniFrac distances between DNA (blue) and RNA (red) components of the microbiome. Top genera are indicated in gray fonts, and their potential roles in driving the different microbiome components are represented by linear lines. **b** Taxonomic profiles of DNA and RNA components of the microbiome. The top 15 genera based on overall relative abundance across the dataset are presented, with the remaining genera and those without genus-level taxonomic classification grouped as “minor/unclassified.” **c** DeSeq2 analysis indicating the OTUs significantly enriched (i.e., FDR-corrected *p* < 0.05) in the DNA (blue) and RNA (red) components of the indoor air microbiome. Genus-level classification are provided for each OTU where available. All analyses showed that the genetic pool (DNA, blue) was strongly characterized by environmental genera that were less likely to be metabolically active, whereas host-associated genera characterized the viable population (RNA)

Air samples in a residential setting were collected to compare the DNA (genetic pool) and RNA (metabolic pool, viable) components of the indoor air microbiome. To our understanding, this is the first account involving DNA and RNA fractions of the of indoor air and surface microbiomes. Similar to a previous work of outdoor air [21], the genetic and the metabolic pool were significantly different in both community membership (unweighted UniFrac Global ANOSIM 0.720, PERMANOVA pseudo-F 8.57, both *p* = 0.001) and community composition (weighted UniFrac Global ANOSIM 0.999, PERMANOVA pseudo-*F* = 56.4, both *p* = 0.001). Environmental genera drove the differentiation of the genetic pool, whereas host-associated genera drove the differentiation of the viable population (Fig. 4a). Within each of the DNA and RNA fractions, communities did not significantly differ between extraction time points and within replicates within time points (FDR-adjusted *p* > 0.05 for ANOSIM and PERMANOVA and unweighted and weighted UniFrac). The differences in the taxonomic profiles of the DNA and RNA populations were also consistent with the community composition data (Fig. 4b). Specifically, the majority of taxa present in the DNA pool belonged to those of environmental origins including *Sphingomonas* and *Porphyrobacter*. In contrast, the taxa detected in the RNA population originated predominantly from humans. The underrepresentation of environmental genera in the RNA population may suggest that some of the environmental microorganisms present in indoor air are less likely to be metabolically active. However, from results of this study alone, it cannot be confirmed whether these organisms were undergoing a dormant state and have the potential to becomes more metabolically active under more favorable conditions. Dormant members act as genetic seed banks that may be indispensable for maintaining microbial diversity and thus community adaptability under changing environmental conditions [59]. Therefore, despite its presumptive inactivity, their potential contribution to the greater microbial population should not be overlooked. Future longitudinal analyses will be able to address whether these potentially dormant taxa could be resuscitated and bloom under different indoor conditions [60].

DeSeq2 was performed to determine differential abundance of specific OTUs between the genetic and the metabolic pool (Fig. 4c and Additional file 6: Table S4). OTUs that were more abundant in the genetic pool generally are of environmental origins, including OTUs of the *Sphingomonadales* order. While members of *Sphingomonas* and *Sphingopyxis* have been identified in air and on surfaces of different BEs [27, 37, 61], here, we suggest that these taxa were perhaps less likely to be metabolically active in this residence. Conversely, an OTU classified as *Deinococcus* is the most differentially abundant in the viable population, which is consistent with this genus’ ability to be resistant to radiation and desiccation, and survive in harsh indoor environments [12]. OTUs classified as genera associated with humans (*Streptococcus*, *Corynebacterium*, *Staphylococcus*) were also significantly and differentially abundant in the viable populations. Given that some of these genera include potentially pathogenic species, species- and strain-level analyses of the RNA population within indoor air may be warranted, as potentially live and pathogenic microbes may be transmitted between indoor individuals [62, 63].

**Fig. 5:**
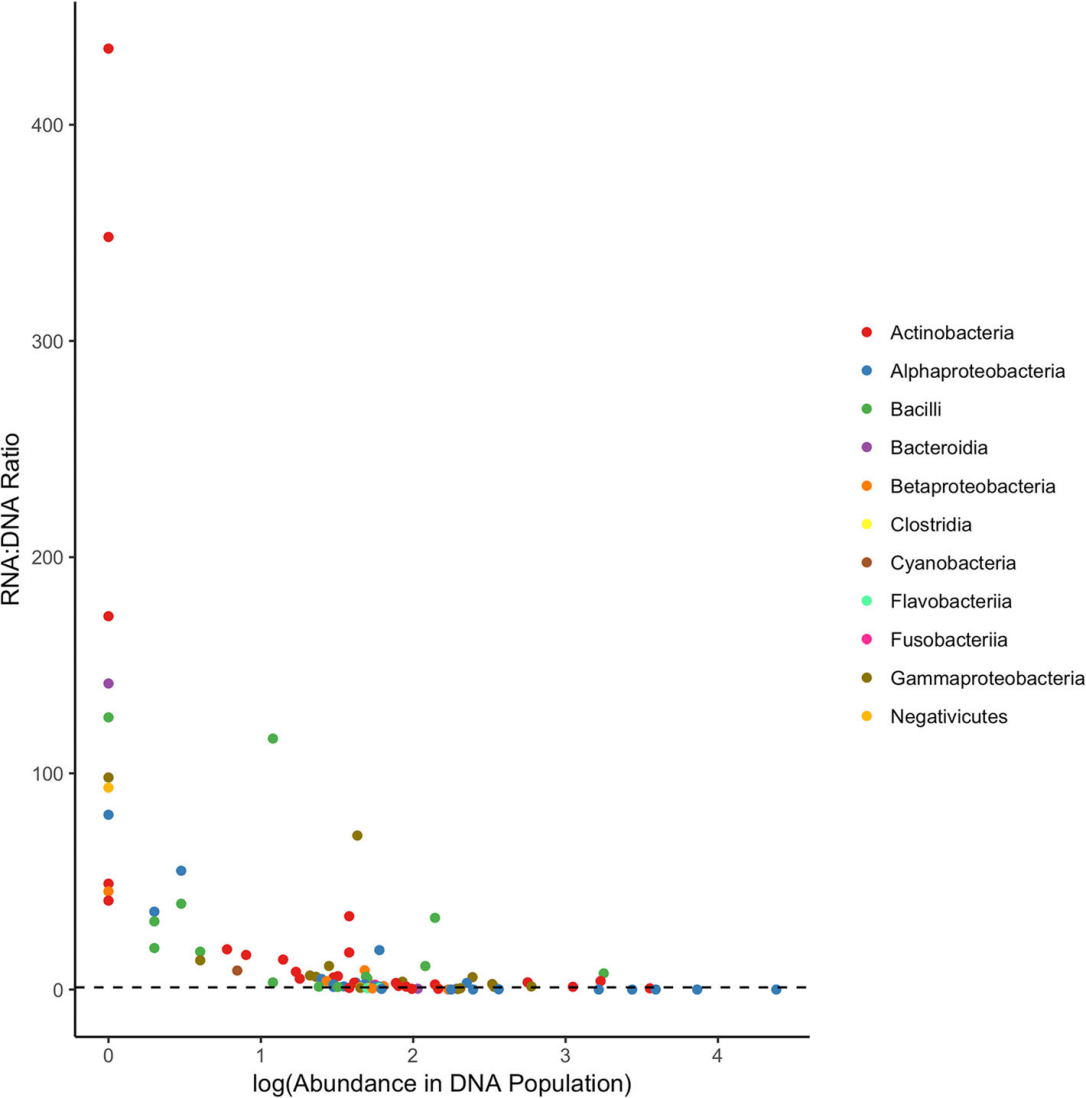
RNA:DNA ratio for OTUs detected plotted against the relative abundance of that OTU in the DNA component of the microbiome. Each point represents an OTU colored at the class taxonomic level. RNA:DNA ratio calculated based on the relative abundances of that particular OTU in their respective RNA and DNA communities. Horizontal dotted black line represents a ratio of 1. Rare taxa based in the DNA-based communities showed a higher metabolic potential

The RNA:DNA abundance ratios for OTUs present in both DNA and RNA populations were calculated to estimate their metabolic potentials. The ratios for these OTUs ranged from 0.002 to over 400 (Fig. 5 and Additional file 7: Table S5). Similar to outdoor air [21], OTUs with higher ratios were those considered to be more rare in the genetic pool. The ratio is strongly and negatively correlated with the relative abundance within the genetic pool (Spearman’s correlation *r* = − 0.764, *p* < 0.0001). OTUs with high RNA:DNA ratios include members of *Microlunatus*, a genus previously postulated to be a rare but active in cleanroom surface microbiome [12]. Consistent with the DeSeq2 result, OTU_60 of *Brachybacterium* had an RNA:DNA ratio at over 170. Different species of *Brahchybacterium* collected over multiple seasons from bioaerosols of Chinese residences had previously been demonstrated to be culturable, suggesting that members of this genus can remain viable in indoor air [64]. In addition, taxa belonging to *Psychrobacter* and *Veillonella*, both of which have been detected previously in low levels in bioaerosols of different BEs [36, 65, 66], had RNA:DNA ratios of almost 100. Interestingly, different OTUs of the same genera may have high or low RNA:DNA ratios depending on the taxon (e.g., OTU_880, OTU_37, and OTU_208 within *Pseudomonas*), which may suggest species or strain-level variations in activity that may or may not have ecological, physiological, or clinical importance [67, 68]. Also consistent with the DeSeq2 results, OTUs of the *Sphingomonadales* (OTU_5, OTU_12, and OTU_21) had the lowest RNA:DNA ratios, further suggesting that these abundant members have lower metabolic potentials in the environment sampled.

Network analysis was performed respectively for the genetic and the metabolic pool of the community (Additional file 8: Figure S1 and Additional file 9: Table S6). For both networks, taxa involved in significant associations are not necessarily those classified as the abundant genera, as suggested by the majority of taxa classified into genera grouped into the “minor/unclassified” group. OTU_100 of *Prevotella* is represented as a hub population, being significantly associated with five other taxa in the DNA-based network. In contrast, hub population was not observed for the RNA-based network. Genera with high metabolic potential as inferred by RNA:DNA ratio (OTU_29 of *Microlunatus*, OTU_72 of *Psychrobacter*, and OTU_50 of *Veillonella*) also presented OTUs that were significantly associated with other taxa in the viable network. Significant and positive correlations (i.e., co-occurrence associations) within members of the same genus were only observed in the DNA population, whereas OTUs of different genera could be involved in both co-occurrence and negative (i.e., co-exclusion) associations in the DNA and RNA-based networks (Additional file 10: Figure S2a and b). Despite the variation in the taxa involved in the respective association networks, network structural properties, as portrayed by network degree distribution (Additional file 10: Figure S2c) and natural connectivity in response to random (Additional file 10: Figure S2d) or non-random node removal (Additional file 10: Figure S2e and f) appear to be similar between the networks. While structural properties between the DNA and RNA-based networks were similar, the difference in taxa involved in the respective networks questions the significance of using DNA data for microbial network analyses. Indeed, results obtained using RNA data from the viable communities may provide a more direct inference of potential ecological associations between community members, by removing any potential noise from DNA data derived from non-active or non-viable microorganisms.

Source-tracking analysis was performed to estimate how viable populations of nearby surface sources potentially contributed to the air sink viable communities and vice versa. In general, the surface closest to the air samplers (i.e., wooden bedside rim) showed approximately 50% community overlap with the air communities (Table 1). Interestingly, a significant distance-decay effect was observed when surface microbiomes were analyzed as sources to the air viable community (Kendall’s τ = − 0.478, *p* = 0.0001), which may be possible as a result of resuspension of surface microbiomes (some of these microbes may be active) from the resident into the adjacent air [69, 70], despite the low occupancy of the residence. However, the mechanisms of any possible resuspension of active microbes from indoor surface to the air will require additional analyses in controlled chamber environments, such as those performed testing the relationships between genetic pools of indoor occupants and indoor [71, 72]. Nonetheless, to our understanding, this is the first demonstration of a distance effect within the viable community between air and surface media within a single room of a BE. Given that microbiomes between indoor media (which can also encompass indoor occupants) may be closely connected [37, 40, 63, 73], understanding the roles of transfers of active microbes between these media, and the conditions that promote such transfers, may be beneficial in paths towards ultimately creating a suitable indoor environment to minimize active pathogen transmission and maximize occupant health.

**Table 1:** SourceTracker predictions for proportions of potential sources and sinks between air and surfaces sampled

Source ecosystem	Sink ecosystem	Source and sink distance (cm)	Source proportion (%)
Air			
Air	Bed rim (bedside)—wood	30	49.5
Air	Chair seat—leather	90	0
Air	Bed rim (bedfront)—wood	180	11.0
Air	Window-side (indoor)—stone	190	12.8
Air	Window-side (outdoor)—cement	210	0
Surface–material			
Bed rim (bedside)—wood	Air	30	52.2
Chair seat-leather	Air	90	0.01
Bed rim (bedfront)—wood	Air	180	0
Window-side (indoor)—stone	Air	190	1.4
Window-side (outdoor)—cement	Air	210	0

In summary, our residential analysis revealed strong variations in DNA and RNA components of the residential microbiomes in terms of community structures, taxonomies, and associative networks. As our in vitro tests suggested, methodological variations may have contributed to the observed DNA and RNA community differences. However, the increased abundance of host-associated taxa in the RNA population in our household analysis is not biologically improbable. Skin shedding, and talking and coughing from indoor occupants introduce host-associated organisms that may persist and remain viable and/or active for extended periods of time onto BE surfaces and into the air [4, 73, 74]. Conversely, environmental taxa that appeared to be more abundant in the DNA population may have been carried over long distances from the outdoors. *Sphingomonas*, OTUs of which were overrepresented in DNA population and classified as having low RNA:DNA ratios in our residence bioaerosols, are commonly detected in BE samples, both in the air [27, 37, 61] and as biofilms on surfaces [75–77], which may be their preferred mode of survival in BEs.

Estimating metabolic potential by calculating the genetic pool to viable population ratio has been reported across ecosystems [21, 78, 79]. However, careful interpretation of ratio estimates is important. RNA:DNA ratios can vary between and within populations in different life stages [15] and are dependent on the sampling depth [80]. Specifically, based on models by Steven et al. [80], some active taxa may be classified as dormant members within a mixed community although the reverse, where a dormant taxa is misclassified as active, was found to be much less common. Dormant cells may accumulate high numbers of ribosomes appearing as active organism through RNA:DNA ratios [15], though it is also possible that microorganisms with low energy output metabolism would appear as dormant [35]. Many of these RNA:DNA ratio limitations come as the consequence of the inappropriate inferences of metabolic activity through rRNA sequence analysis [16], but could be minimized if the rRNA was primarily used as a proxy for viability. Given that the majority of DNA detected in BEs may originate from non-viable cells [11], DNA-based microbial community analysis will likely skew the results towards taxa that are not contributing to relevant ecosystem processes.

## Conclusions

This study confirms the high level of technical variability, similar for both DNA and RNA sample processing, and emphasizes the relevance of replicates in molecular-based microbial community studies. Although the FastPrep method of DNA/RNA co-extraction had the highest efficiency of RNA recovery, the overall differences among the methods did not rise to the level of a strong recommendation of one method exclusively. Similarly, no recommendations can be made for sample swab methods due to the similarity of their performance. Flash freezing with liquid N_2_ was the preferred method for long-term sample storage, although strikingly, significant nucleic acid degradation was noticed in all storage methods by the first week. Despite the potential logistical difficulties, our strongest recommendation for minimizing technical biases is to perform the nucleic acid extraction within the first week, and then store the nucleic acids in preservation solutions until further analysis.

In spite of the inherent technical biases, the biological variation observed in activity/viability of residential BE samples in this study highlighted the potential roles of key microbial taxa. Specifically, we identified taxa that by DNA-based sequence analysis appeared to be in high abundance, but by rRNA-based sequence analysis suggestive of dormancy or non-viability. Conversely, we identified low-abundance taxa that by rRNA-based sequencing may have important ecosystem functions. Further studies are required to fully demonstrate and understand the effectiveness of rRNA as a proxy for viability, but certainly rRNA-based microbial community studies offer a new dimension of information not accessible by the DNA-based analysis. It is our opinion that future nucleic acid-based BE studies incorporate rRNA preparation and analysis to provide insight into microbial population dynamics of the active/viable taxa [15], and to determine how BE factors drive microbiome structure and ultimately affect occupant health.

## Additional files

Additional file 1: **Table S1.** Pure strains and primers used in this study. List of the pure strains and primers used in this study and their references. (DOCX 123 kb)

Additional file 2: **Table S2.** Nucleic acids processing protocols. qPCR and retrotranscription reactions and conditions. (DOCX 65 kb)

Additional file 3: **Table S3.** Sampling, extraction, and preservation products and surfaces tested. List of in vitro tests and the products tested, including the manufacturer details. (DOCX 77 kb)

Additional file 4: **Text S1.** Detailed description of optimized DNA/RNA co-extraction protocols. Step by step detailed co-extraction protocols. Steps modified from the manufacturers’ instructions specified. (DOCX 18 kb)

Additional file 5: **Text S2.** Detailed protocols of sample storage test. (DOCX 64 kb)

Additional file 6: **Table S4.** Differentially abundant OTUs and their taxonomic classification between DNA and RNA populations in residential air microbiome. (XLSX 45 kb)

Additional file 7: **Table S5.** RNA:DNA ratio of shared OTUs in residential air microbiome. (XLSX 37 kb)

Additional file 8: **Figure S1.** Network analysis of DNA and RNA components of the microbiome. Each node represents a particular OTU that is involved in significant correlation with other OTU(s) as calculated in SPIEC-EASI. Networks generated using Cytoscape. OTUs are colored by their genera and are connected to other OTUs to represent positive (blue edges) or negative (red edges) correlations. The strength of the correlation is represented by the thickness of the edge. Hub OTUs, and OTUs with high metabolic potential, are indicated with their OTU number and genus-level taxonomy. Taxa with significant associations are not necessarily classified as the abundant. Similar structure properties were detected for both network analyses (Additional file 10: Figure S2), although the taxa involved and their correlations differed. (PDF 5496 kb)

Additional file 9: **Table S6.** OTUs and their genus-level classification involved in significant associations in the active and/or total populations. (XLSX 47 kb)

Additional file 10: **Figure S2.** Correlation network structure between DNA and RNA components of the microbiome. Density plot of intra-genus (red) and inter-genus (blue) correlations within the (A) DNA and (B) RNA components of the microbiome. Plots are faceted for each microbiome component based on whether the correlation is positive or negative. C–F. Network structure comparison between DNA (blue) and RNA (red) components of the microbiome in terms of (C) degree distribution, and natural connectivity upon removal of network node either (D) randomly or via decreasing order of (E) degree or (F) betweenness centrality. Network structure analyzed using SPIEC-EASI. (PDF 1413 kb)

## Abbreviations

BE: Built environment
cDNA: Complementary DNA
FDR: False discovery rate
gDNA: Genomic DNA
HTS: High-throughput sequencing
KW: Kruskall-Wallis
MW: Mann-Whitney
OTU: Operational taxonomic unit
PBS: Phosphate-buffered saline
QIIME: Quantitative Insights Into Microbial Ecology
qPCR: Quantitative polymerase chain reaction
rRNA: Ribosomal RNA
SPIEC-EASI: SParse InversE Covariance Estimation for Ecological Association Inference
