# Supplementary material for: A comparison of methods used to unveil the genetic and metabolic pool in the built environment
Source: Microbiome. 2018 Apr 16;6:71. doi: 10.1186/s40168-018-0453-0 (PMC5902888; doi:10.1186/s40168-018-0453-0)
Supplement: Supplementary file 1 — Table S1. Pure strains and primers used in this study. List of the pure strains and primers used in this study and their references. (DOCX 123 kb) [file 40168_2018_453_MOESM1_ESM.docx]

**Additional file 1: Table S1. Pure strains and primers used in this study.** List of the pure strains and primers used in this study and their references.

| **Pure strains** | **Growth media** | **Type of cell** | **qPCR Primer sequences (5' - 3')** | | **Region** | **Reference** |
| --- | --- | --- | --- | --- | --- | --- |
| *P. syringae* | LB | Gram negative | Psyr_74f: | GGGTACTTGTACCTGGTGGC | 16S rRNA | this study |
| B728a [1] |  | Bacteria | Psyr_152r: | CGAGCGTTATCCCCCACTAC |  |  |
| *B. subtilis* | LB | Gram positive, | Bsub_1046f: | CAGAGTGACAGGTGGTGCAT | 16S rRNA | this study |
| PY79-GFP [2] |  | Bacteria | Bsub_1532r: | CACCTTCCGATACGGCTACC |  |  |
| *S. cerevisiae* | YPD | Unicellular fungi, | Scer_1109f: | GCCCCGTTGGAAATCCAGTA | 18S rRNA | this study |
| ATCC 201388 [3] |  | Yeast | Scer_1313r: | GGCCCATTCGGGTCTTGTAA |  |  |
| *A. nidulans* | 5YEG | Multicellular | Anid_112f: | CCCCTCCAGAGTGAATACCT | 18S rRNA | this study |
| FGSC A1416 [4] |  | filamentous fungi | Anid_405r: | CCGAAGTCGGGGTTTTTAGC |  |  |
|  |  | **Taxa** | **HTS Primer sequences (5' - 3')** | | **Region** | **Reference** |
|  |  | Bacteria | 16S_515*fw* | GTGCCAGCMGCCGCGGTAA | 16S rRNA, V4 | [5] |
|  |  |  | 16S_806*rv* | GGACTACHVGGGTWTCTAAT |  |  |
|  |  | Fungi | ITS1_18Sfw | GTAAAAGTCGTAACAAGGTTTC | ITS 1 | [6] |
|  |  |  | ITS1_5.8S*rv* | GTTCAAAGAYTCGATGATTCAC |  |  |

**Additional file 1: Table S1 references:**

1. Miller WG, Leveau JHJ, Lindow SE. Improved gfp and inaZ Broad-Host-Range Promoter-Probe Vectors. Mol Plant Microbe Interact. 2000;13:1243–50.

2. Rosenberg A, Sinai L, Smith Y, Ben-Yehuda S. Dynamic expression of the translational machinery during *Bacillus subtilis* life cycle at a single cell level. PLoS ONE. 2012;7:e41921.

3. Brachmann CB, Davies A, Cost GJ, Caputo E, Li J, Hieter P, et al. Designer deletion strains derived from *Saccharomyces cerevisiae* S288C: a useful set of strains and plasmids for PCR-mediated gene disruption and other applications. Yeast Chichester Engl. 1998;14:115–32.

4. Morozov IY, Jones MG, Spiller DG, Rigden DJ, Dattenböck C, Novotny R, et al. Distinct roles for Caf1, Ccr4, Edc3 and CutA in the co-ordination of transcript deadenylation, decapping and P-body formation in *Aspergillus nidulans*. Mol Microbiol. 2010;76:503–16.

5. Leung MHY, Wilkins D, Li EKT, Kong FKF, Lee PKH. Indoor-air microbiome in an urban subway network: diversity and dynamics. Appl Env Microbiol. 2014;80.

6. Findley K, Oh J, Yang J, Conlan S, Deming C, Meyer JA. Topographic diversity of fungal and bacterial communities in human skin. Nature. 2013;498.
