# Supplementary material for: A comparison of methods used to unveil the genetic and metabolic pool in the built environment
Source: Microbiome. 2018 Apr 16;6:71. doi: 10.1186/s40168-018-0453-0 (PMC5902888; doi:10.1186/s40168-018-0453-0)
Supplement: Supplementary file 2 — Table S2. Nucleic acids processing protocols. qPCR and retrotranscription reactions and conditions. (DOCX 65 kb) [file 40168_2018_453_MOESM2_ESM.docx]

**Additional file 2: Table S2. Nucleic acids processing protocols.** qPCR and retrotranscription reactions and conditions.

| **qPCR reaction protocol** | | | | | | |
| --- | --- | --- | --- | --- | --- | --- |
|  | **Reagents:** | **Final concentration:** | **Quantification conditions:** | | | |
|  | ExTaq Buffer^1^ | 1X | Initial denaturalization | 95°C, 5 min. | |  |
|  | dNTPs mix^1^ | 200μM | Amplification | 95°C, 30 sec. | |  |
|  | TaKaRa ExTaq^1^ | 0.025U/μL |  | 60°C, 30 sec. | | 40 cycles |
|  | Primers | 150nM of each |  | 72°C, 30 sec. | |  |
|  | BSA | 1μg/μL | Melting curve | 60- 95°C, 10 sec. | |  |
|  | EvaGreen | 0.2X |  |  | |  |
|  | Final volume | 25ul | ^1^ Takara Bio Inc., Otsu-Shi, Japan | | | |
| **Retrotranscription protocol** | | | | | | |
|  | **Reagents:** | **Final concentration:** | **Quantification conditions:** | | | |
|  | First-Strand Buffer^2^ | 1X | Initial RNA straightening | | 65°C, 5 min. | |
|  | DTT^2^ | 500nM | Retrotranscription | | 55°C, 60 min. | |
|  | SuperScript III RT^2^ | 10U/μl |  | | | |
|  | dNTPs mix | 200μM |  | | | |
|  | Primers | 200μM |  | | | |
|  | Final volume | 20μl | ^2^Invitrogen, Thermo Fisher Scientific, Waltham, MA, USA | | | |
