# Supplementary material for: A comparison of methods used to unveil the genetic and metabolic pool in the built environment
Source: Microbiome. 2018 Apr 16;6:71. doi: 10.1186/s40168-018-0453-0 (PMC5902888; doi:10.1186/s40168-018-0453-0)
Supplement: Supplementary file 3 — Table S3. Sampling, extraction, and preservation products and surfaces tested. List of in vitro tests and the products tested, including the manufacturer details. (DOCX 77 kb) [file 40168_2018_453_MOESM3_ESM.docx]

**Additional file 3: Table S3. Sampling, extraction, and preservation products and surfaces tested.** List of *in vitro* tests and the products tested, including the manufacturer details.

| **Type of test** | **Items tested** | **Manufacturer** |
| --- | --- | --- |
| Kit tested | **Power protocol:** DNeasy PowerSoil Kit & PowerMicrobiome RNA Isolation Kit | Qiagen, Hilden, Germany |
|  | **AllPrep protocol:** AllPrep DNA/RNA Mini Kit | Qiagen, Hilden, Germany |
|  | **FastPrep protocol:** FastDNA & FastRNA Spin kit for Yeast | MP Biomedicals, Solon, OH, USA |
| Swab tested | eSwab | Copan Diagnostics, Corona, CA, USA |
|  | BBL CultureSwab EZ | Becton, Dickinson and Company, Sparks, MD, USA |
|  | BiSKit | QuickSilver Analytics, Abingdon, MD, USA |
| Surface tested | Untreated wood |  |
|  | Aluminum tray |  |
|  | Plastic tray |  |
| Sample storage methods tested | RNAlater (at -20°C & -80°C) | Thermo Fisher Scientific, Waltham, MA, USA |
|  | RNAProtect | Qiagen, Hilden, Germany |
|  | LifeGuard Preservation Solution | Qiagen, Hilden, Germany |
|  | Formamide | Thermo Fisher Scientific, Waltham, MA, USA |
|  | Liquid nitrogen |  |
| Extracted RNA storage solution | RNAStable LD | Biomatrica, San Diego, CA, USA |
