# Supplementary material for: A comparison of methods used to unveil the genetic and metabolic pool in the built environment
Source: Microbiome. 2018 Apr 16;6:71. doi: 10.1186/s40168-018-0453-0 (PMC5902888; doi:10.1186/s40168-018-0453-0)
Supplement: Supplementary file 4 — Text S1. Detailed description of optimized DNA/RNA co-extraction protocols. Step by step detailed co-extraction protocols. Steps modified from the manufacturers’ instructions specified. (DOCX 18 kb) [file 40168_2018_453_MOESM4_ESM.docx]

**Additional file 4: Text S1.** **Detailed description of optimized DNA/RNA co-extraction protocols.** Step by step detailed co-extraction protocols. Steps modified from the manufacturer’s instructions specified.

***A. Power co-extraction***

1. Starting with the DNeasy PowerSoil Kit, add the samples to the PowerBeads Tubes and vortex.

2. Add 60 μL of Solution C1 and vortex.

3. *(modified from manufacturer’s instructions)* Secure the tubes in the FastPrep Instrument (MP Biomedical, Burlingame, CA, USA) and process them at 6 m/s for 30 sec. Places the tube in ice for 1 min., to cool the samples down. Repeat the lysis step in the FastPrep Instrument.

4. Centrifuge tubes at 10,000 × g for 30 sec. at room temperature. Transfer the all supernatant to a clean tube.

5. Add 250 μl of Solution C2 and vortex for 5 sec. Incubate at 4°C for 5 min.

6. Centrifuge the tubes at room temperature for 1 min. at 10,000 × g. Transfer the all supernatant to a clean tube.

7. Add 200 μl of Solution C3 and vortex briefly. Incubate at 4°C for 5 min.

8. Centrifuge the tubes at room temperature for 1 min. at 10,000 × g. Transfer the all supernatant to a clean tube.

9. *(modified from manufacturer’s instructions*) Shake to mix Solution C4 before use. Add 3200 μl of Solution C4 to the supernatant and vortex for 5 sec.

10. *(modified from manufacturer’s instructions*) Load 700 μL of sample into the DNA Spin Filter and centrifuge the tubes at 10,000 g for 1 min.

Recover the flow-through in a 15 mL tube in ice containing 1.5 mL of PM3 from the PowerMicrobiome RNA Isolation Kit and 1 volume of ethanol 100% (for further RNA extraction).

Repeat the step until all the sample is being passed through the Spin Filter. Reserve the DNA Spin Filter at 4C for further gDNA purification after the RNA purification is being complete.

11. *(modified from manufacturer’s instructions*) Proceed with the RNA extraction first using the PowerMicrobiome RNA Isolation Kit: Load the sample from the 15 mL tube into the RNA Spin Filter and centrifuge at 13,000 × g for 1 min. Discard the flow-through.

12. Shake the solution PM5 and add 650 μL to the RNA Spin Filter. Centrifuge at 13,000 × g for 1 min.

13. Discard the flow-through and centrifuge again at 13,000 × g for 1 min. to remove the residual wash.

14. *(modified from manufacturer’s instructions*) Transfer the RNA Spin Filter to a clean tube and add 17 μL of RNase-free, ensuring to wet all the membrane, and let stand for 1 min. Then centrifuge at 13,000 × g for 1 min., and pass the same flow-through again through the membrane to recover all the RNA.

15. *(modified from manufacturer’s instructions*) With the eluted RNA, proceed to the gDNAse digestion.

16. *(modified from manufacturer’s instructions*) Continue with the gDNA extraction using again the DNeasy PowerSoil Kit. Add 500 μL of Solution C5 to the DNA Spin Filter reserved at 4°C.

17. Centrifuge at 10,000 × g 30 sec. and discard the flow-through.

18. Centrifuge at 10,000 × g 1 min. to get remove the residual wash.

19. *(modified from manufacturer’s instructions*) Transfer the gDNA Spin Filter to a new tube. Elute the gDNA in 40 μL of DNA-free water, ensuring to wet all the membrane, and let stand for 1 min. Centrifuge at 10,000 × g 30 sec. Pass the same flow-through again through the membrane to ensure recovering all the gDNA. The gDNA is ready for further use. Preserve it at -20°C.

***B. AllPrep co-extraction***

1. *(modified from manufacturer’s instructions*) Prepare the 15 mL lysis tubes adding 1g of carbide beads 0.25mm (MO BIO Laboratories Inc., Carlsbad, CA), 700 μL of RTL Plus Buffer (part of the AllPrep DNA/RNA Mini Kit; Qiagen, Hilden, Germany), and 7 μL of beta-mercapthethanol (Sigma Aldrich, St. Louis, MO, USA). Add the samples to the lysis tubes.

2. *(modified from manufacturer’s instructions*) Secure the tubes in the FastPrep Instrument (MP Biomedical, Burlingame, CA, USA) and process them at 6 m/s for 30 sec. Places the tube in ice for a min., to cool the samples down. Repeat the lysis step in the FastPrep Instrument.

3. Transfer 700 μL of the sample to a AllPrep DNA spin column and centrifuge 30 sec. at 10,000 × g. Recover the flow-through in 1 volume of ethanol 100% in ice, and mix it. Repeat the step until all the sample pass through the AllPrep DNA spin column.

4. Transfer the AllPrep DNA spin column to a clean tube and reserve them at 4°C for further gDNA purification after the RNA purification is being complete.

5. Proceed directly to the RNA extraction: Transfer 700 μL the sample to a RNeasy spin column and centrifuge 15 sec. at 10,000 × g. Discard the flow-through. Repeat until all the sample pass through the RNeasy spin column.

6. Add 700 μL of Buffer RW1 and centrifuge 15 sec. at 10,000 × g. Discard the flow-through.

7. Add 500 μL of Buffer RPE and centrifuge 15 sec. at 10,000 × g. Discard the flow-through. Repeat the step.

8. Transfer the RNeasy spin column to a new collection tube and centrifuge 1 min. at 10,000 × g.

9. *(modified from manufacturer’s instructions*) Transfer the RNeasy spin column to a new tube. Add 17 μL of RNase-free, ensuring to wet all the membrane, and let stand for 1 min. Then centrifuge at 10,000 × g for 1 min., and pass the same flow-through again through the membrane to recover all the RNA.

10. *(modified from manufacturer’s instructions*) With the eluted RNA, proceed to the DNase digestion.

11. Add 500 μL of Buffer AW1 to the AllPrep DNA spin column reserved at 4°C. Centrifuge 15 sec. at 10,000 × g. Discard the flow-through.

12. Add 500 μL of Buffer AW2 and centrifuge 2 min. at 10,000 × g. Discard the flow-through.

13. Centrifuge 1 min. at 10,000 × g.

14. *(modified from manufacturer’s instructions*) Transfer the column to a new tube. Elute the gDNA in 40 μL of DNA-free water, ensuring to wet all the membrane, and let stand for 1 min. Centrifuge at 10,000 × g 30 sec. Pass the same flow-through again through the membrane to ensure recovering all the gDNA. The gDNA is ready for further use. Preserve it at -20°C.

***C. FastPrep co-extraction***

1. Starting with the FastRNA Spin kit for Yeast, add the samples to the Lysis Matrix Y and add 800 μL of FastRNA Lysis Buffer.

2. *(modified from manufacturer’s instructions*) Secure the tubes in the FastPrep Instrument (MP Biomedical, Burlingame, CA, USA) and process them at 6 m/s for 30 sec. Places the tube in ice for a min., to cool the samples down. Repeat the lysis step in the FastPrep Instrument.

3. Centrifuge the sample 1 min. at 12,000 × g.

4. *(modified from manufacturer’s instructions*) Transfer 700 μL of the supernatant to a FastRNA Spin Column 1. Centrifuge 30 sec. at 8,000 × g. Recover the flow-through in a 15 mL tube with 1 volume of ethanol 100% in ice. Repeat the step until all the sample pass through the FastRNA Spin Column 1.

5. Reserve the FastRNA Spin Column 1 at 4°C for further gDNA purification after the RNA purification is being complete.

6. Transfer 650 μL of the sample to the FastRNA Spin Column 2. Centrifuge 30 sec. at 12,000 × g. Recover the flow-through in a 15 mL tube in ice for further gDNA purification after the RNA purification is being complete. Repeat the step until all the sample pass through the FastRNA Spin Column 2.

13. Add 400 μL of FastRNA Prep Buffer to the FastRNA Spin Column 2 and centrifuge 1 min. at 12,000 × g. Discard the flow-through.

14. Add 800 μL of FastRNA Wash Buffer to the FastRNA Spin Column 2 and centrifuge 30 sec. at 12,000 × g. Discard the flow-through. Repeat the step adding 400 μL of FastRNA Wash Buffer.

15. Transfer the FastRNA Spin Column 2 to a new collection tube and centrifuge 2 min. at 12,000 × g.

17. Transfer the FastRNA Spin Column 2 to a new tube Add 17 μL of RNase-free, ensuring to wet all the membrane, and let stand for 1 min. Then centrifuge at 10,000 × g for 1 min., and pass the same flow-through again through the membrane to recover all the RNA.

18. *(modified from manufacturer’s instructions*) With the eluted RNA, proceed to the DNase digestion.

19. Add 100 μL of DNA-free water into the FastRNA Spin Column 1 reserved at 4°C. Let it stand for 1 min. and centrifuge at 8,000 × g 1 min.

20. Recover the flow-through along with the rest of the flow-through preserved in the 15 mL tube in ice from step 6. Discard the FastRNA Spin Column 1.

21. *(modified from manufacturer’s instructions*) Continue with the FastDNA Spin kit: Add 800 μL of Binding Matrix to the 15 mL tube. Incubate in a rotator for 5 min. at room temperature.

22. Transfer 700 μL of the sample to a SPIN Filter and centrifuge 1 min. at 14,000 × g. Discard the flow-through. Repeat the step until all the sample pass through the SPIN Filter. Note: Mix the sample before transfer to the SPIN Filter.

23. Add 500 μL of SEWS-M resuspending the pellet pipetting carefully up and down. Centrifuge 1 min. at 14,000 × g. Discard the flow-through. Repeat the centrifugation during 2 min.

24. *(modified from manufacturer’s instructions*) Transfer the SPIN Filter to a new tube and add 40 μL of DNA-free water and resuspend the Binding Matrix by tapping the tubes. Incubate the tubes at 55°C for 5 min. in a heat block. Centrifuge the tubes 1 min. at 14,000 × g. The gDNA is ready for further use. Preserve it at -20°C.
