# Supplementary material for: A comparison of methods used to unveil the genetic and metabolic pool in the built environment
Source: Microbiome. 2018 Apr 16;6:71. doi: 10.1186/s40168-018-0453-0 (PMC5902888; doi:10.1186/s40168-018-0453-0)
Supplement: Supplementary file 5 — Text S2. Detailed protocols of sample storage test. (DOCX 64 kb) [file 40168_2018_453_MOESM5_ESM.docx]

**Additional file 5: Text S2. Detailed protocols of sample storage test.**

- **Liquid nitrogen**: the pellets were flash frozen using liquid nitrogen and samples stored at -80°C.
- **RNAlater:** the pellets were resuspended in 500 μL of RNAlater and incubated at 4°C for 1 hour. Then, the cells were pelleted again, the RNAlater removed, and the samples stored at -20°C and -80°C.
- **RNAProtect:** the pellets were resuspended in 500 μL of RNAProtect and incubated 5 min. at room temperature. Then, the cells were pelleted again, the RNAProtect removed, and the samples stored at -80°C.
- **LifeGuard:** the pellets were resuspended in 500 μL of LifeGuard and stored at -20°C. The cells were pelleted and the LifeGuard removed before the DNA/RNA co-extractions.
- **Formamide:** the pellets were resuspended in 200 μL of formamide and stored at -20°C. The cells were pelleted and the formamide removed before the DNA/RNA co-extractions.
