# Supplementary figures and images for: A comparison of methods used to unveil the genetic and metabolic pool in the built environment
Source: Microbiome. 2018 Apr 16;6:71. doi: 10.1186/s40168-018-0453-0 (PMC5902888; doi:10.1186/s40168-018-0453-0)

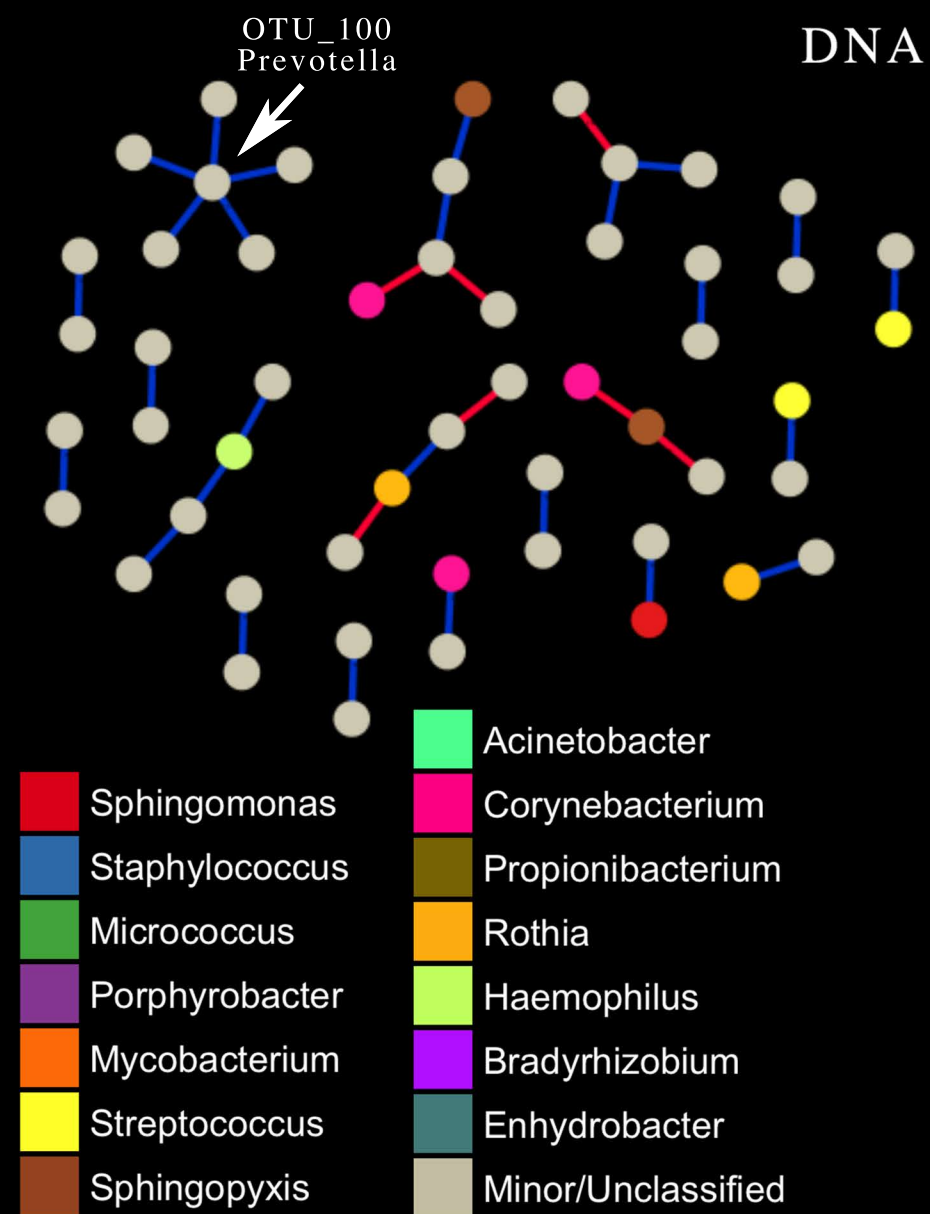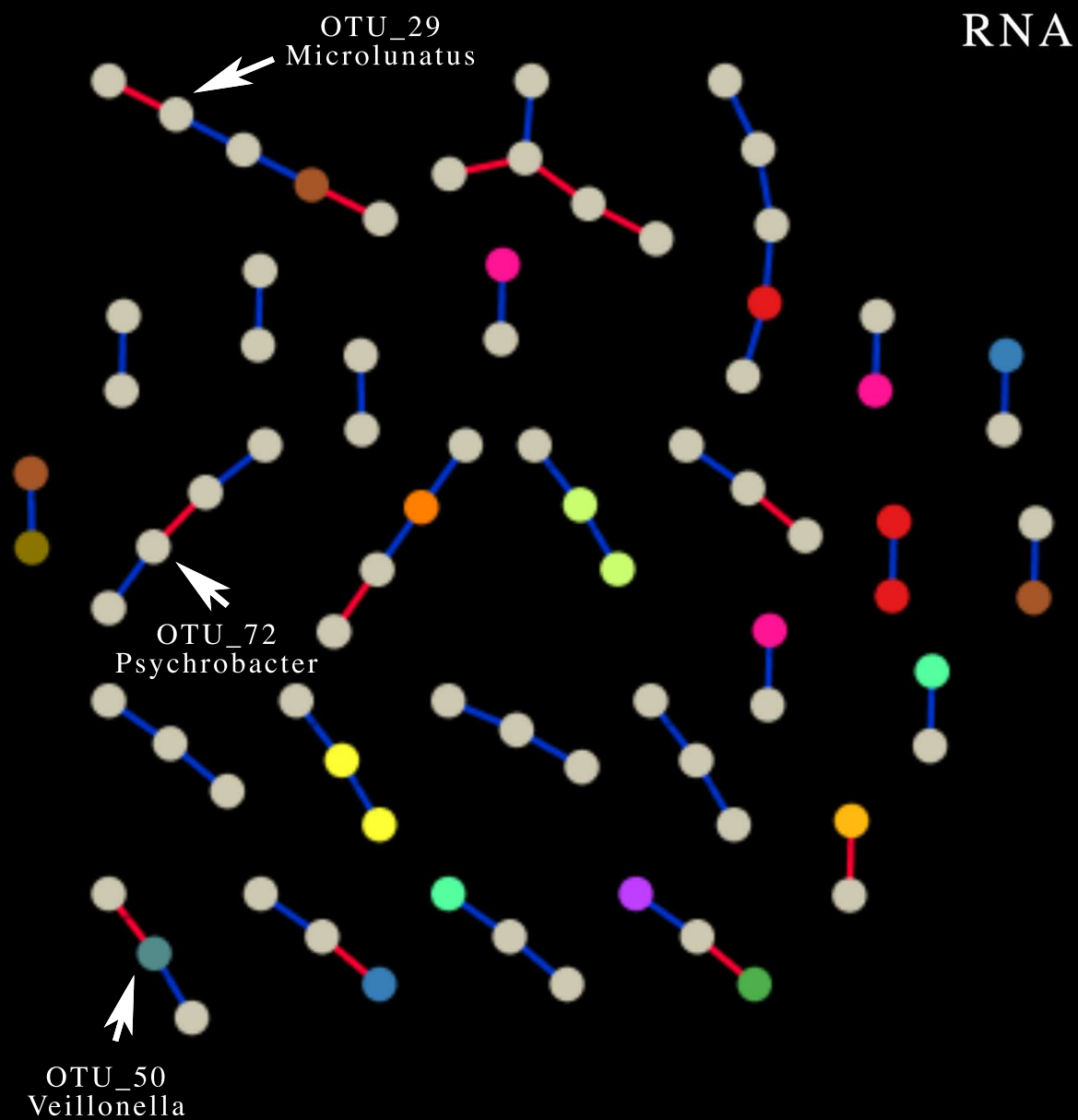

Supplement: Supplementary file 8 — Figure S1. Network analysis of DNA and RNA components of the microbiome. Each node represents a particular OTU that is involved in significant correlation with other OTU(s) as calculated in SPIEC-EASI. Networks generated using Cytoscape. OTUs are colored by their genera and are connected to other OTUs to represent positive (blue edges) or negative (red edges) correlations. The strength of the correlation is represented by the thickness of the edge. Hub OTUs, and OTUs with high metabolic potential, are indicated with their OTU number and genus-level taxonomy. Taxa with significant associations are not necessarily classified as the abundant. Similar structure properties were detected for both network analyses (Additional file 10: Figure S2), although the taxa involved and their correlations differed. (PDF 5496 kb) [file 40168_2018_453_MOESM8_ESM.pdf]
