# Supplementary material for: A comparison of methods used to unveil the genetic and metabolic pool in the built environment
Source: Microbiome. 2018 Apr 16;6:71. doi: 10.1186/s40168-018-0453-0 (PMC5902888; doi:10.1186/s40168-018-0453-0)
Supplement: Supplementary file 10 — Figure S2. Correlation network structure between DNA and RNA components of the microbiome. Density plot of intra-genus (red) and inter-genus (blue) correlations within the (A) DNA and (B) RNA components of the microbiome. Plots are faceted for each microbiome component based on whether the correlation is positive or negative. C–F. Network structure comparison between DNA (blue) and RNA (red) components of the microbiome in terms of (C) degree distribution, and natural connectivity upon removal of network node either (D) randomly or via decreasing order of (E) degree or (F) betweenness centrality. Network structure analyzed using SPIEC-EASI. (PDF 1413 kb) [file 40168_2018_453_MOESM10_ESM.pdf]

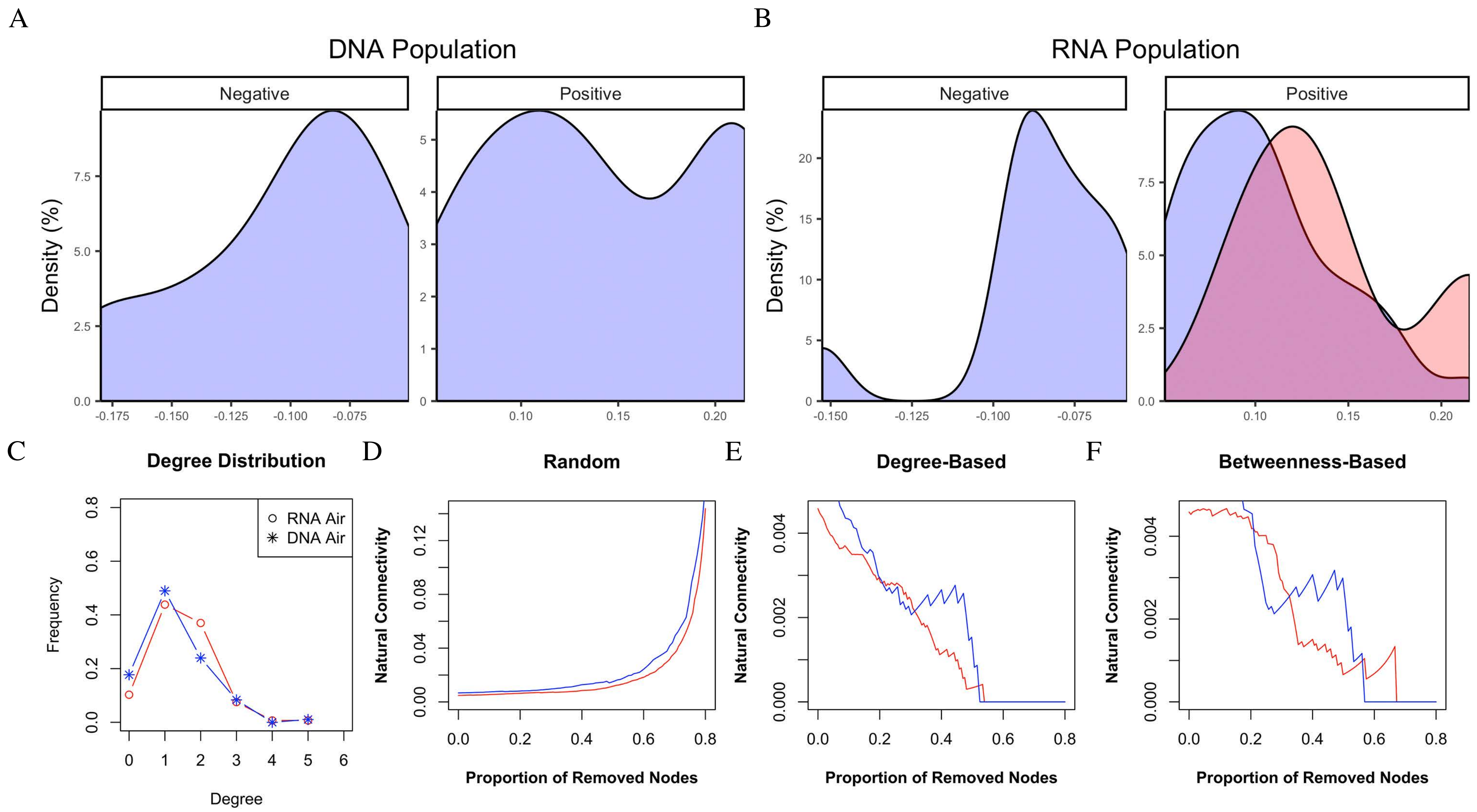


Additional file 10: Figure S2
